# Increasing survival after admission to UK critical care units following cardiopulmonary resuscitation

**DOI:** 10.1186/s13054-016-1390-6

**Published:** 2016-07-09

**Authors:** J. P. Nolan, P. Ferrando, J. Soar, J. Benger, M. Thomas, D. A. Harrison, G. D. Perkins

**Affiliations:** School of Clinical Sciences, University of Bristol, 69 St. Michael’s Hill, Bristol, BS2 8DZ UK; Anaesthesia and Intensive Care Medicine, Royal United Hospital, Combe Park, Bath, BA1 3NG UK; Intensive Care National Audit & Research Centre (ICNARC), Napier House, 24 High Holborn, London, WC1V 6AZ UK; Anaesthesia and Intensive Care Medicine, Southmead Hospital, North Bristol NHS Trust, Bristol, BS10 5NB UK; Faculty of Health and Applied Sciences, Glenside Campus, Blackberry Hill, University of the West of England, Bristol, BS16 1DD UK; Intensive Care Medicine, University Hospitals, Bristol, BS2 8HW UK; Warwick Medical School, University of Warwick, Coventry, CV4 7AL UK; Intensive Care Medicine, Heart of England NHS Foundation Trust, Coventry, CV4 7AL UK

## Abstract

**Background:**

In recent years there have been many developments in post-resuscitation care. We have investigated trends in patient characteristics and outcome following admission to UK critical care units following cardiopulmonary resuscitation (CPR) for the period 2004–2014. Our hypothesis is that there has been a reduction in risk-adjusted mortality during this period.

**Methods:**

We undertook a prospectively defined, retrospective analysis of the Intensive Care National Audit & Research Centre (ICNARC) Case Mix Programme Database (CMPD) for the period 1 January 2004 to 31 December 2014. Admissions, mechanically ventilated in the first 24 hours in the critical care unit and admitted following CPR, defined as the delivery of chest compressions in the 24 hours before admission, were identified. Case mix, withdrawal, outcome and activity were described annually for all admissions identified as post-cardiac arrest admissions, and separately for out-of-hospital cardiac arrest and in-hospital cardiac arrest. To assess whether in-hospital mortality had improved over time, hierarchical multivariate logistic regression models were constructed, with in-hospital mortality as the dependent variable, year of admission as the main exposure variable and intensive care unit (ICU) as a random effect. All analyses were repeated using only the data from those ICUs contributing data throughout the study period.

**Results:**

During the period 2004–2014 survivors of cardiac arrest accounted for an increasing proportion of mechanically ventilated admissions to ICUs in the ICNARC CMPD (9.0 % in 2004 increasing to 12.2 % in 2014). Risk-adjusted hospital mortality following admission to ICU after cardiac arrest has decreased significantly during this period (OR 0.96 per year). Over this time, the ICU length of stay and time to treatment withdrawal has increased significantly. Re-analysis including only those 116 ICUs contributing data throughout the study period confirmed all the results of the primary analysis.

**Conclusions:**

Risk-adjusted hospital mortality following admission to ICU after cardiac arrest has decreased significantly during the period 2004–2014. Over the same period the ICU length of stay and time to treatment withdrawal has increased significantly.

**Electronic supplementary material:**

The online version of this article (doi:10.1186/s13054-016-1390-6) contains supplementary material, which is available to authorized users.

## Background

In a secondary analysis of the Intensive Care National Audit & Research Centre (ICNARC) Case Mix Programme Database (CMPD) undertaken 10 years ago, we determined the characteristics and outcomes for patients admitted to United Kingdom (UK) intensive care units (ICUs) [1]. Since then there have been many developments in post-resuscitation care, which are summarised in guidelines published in 2015 by the European Resuscitation Council and the European Society of Intensive Care Medicine [2, 3]. After very little improvement in cardiac arrest survival rates for three decades [4], several investigators have reported increasing survival following both in-hospital [5] and out-of-hospital cardiac arrest [6]. One study from the United States has documented declining mortality rates among those hospitalised after cardiac arrest in the period 2001–2009 [7]. Two studies, one from Finland (2000–2008) and one from the Netherlands (1999–2009), have documented an improving trend in outcomes after admission to ICU following cardiac arrest [8, 9].

Other studies have shown considerable variation in outcome for post-cardiac arrest patients admitted to ICUs in the United States [10] and Canada [11].

The ICNARC CMPD includes data on admissions to all adult general ICUs in the UK. This facilitates reliable, representative analyses of most patients in the UK admitted to ICU after cardiac arrest. The objective of this study is to investigate trends in patient characteristics and outcome following admission to UK critical care units following cardiopulmonary resuscitation (CPR) for the period 2004–2014 (11 years). Our hypothesis is that there has been a reduction in risk-adjusted (ICNARC model) mortality during 2004–2014. Secondary objectives of our study are to investigate trends in: the proportion of post-cardiac arrest patients having treatment withdrawn and the timing of withdrawal; the lowest temperature of ≤ 34 °C in the first 24 h (as a surrogate for the use of mild induced hypothermia); length of ICU stay among post-cardiac arrest patients; the proportion of post-cardiac arrest patients becoming organ donors.

## Methods

### Case Mix Programme Database

The Case Mix Programme (CMP) is a national comparative audit of adult, general ICUs (including intensive care units and combined intensive care and high dependency units, but not coronary care units) in England, Wales and Northern Ireland co-ordinated by ICNARC. The CMP has received approval from the Patient Information Advisory Group to hold patient identifiable information without consent (approval number PIAG 2-10(f)/2005). Approval by a research ethics committee was not required. Details of the data collection and validation have been reported previously [12].

We undertook a prospectively defined, retrospective analysis of the ICNARC CMPD for the period 1 January 2004 to 31 December 2014. Admissions, mechanically ventilated in the first 24 hours in the critical care unit and admitted following CPR, defined as the delivery of chest compressions in the 24 hours before admission, were identified. Patients sustaining a cardiac arrest in ICU (but not before admission) were excluded from the analysis.

The admissions that were identified were then grouped in the following way:

- **Out-of-hospital cardiac arrest:** location immediately prior to source of admission to the unit as ‘clinic or home’ and source of admission to the unit is ‘A&E, same hospital’.
- **In-hospital cardiac arrest:** location immediately prior to source of admission to the unit is not ‘clinic or home’ and source of admission to the unit is not ‘A&E, same hospital’.

#### Case mix

Age, gender, year of admission and past medical history were extracted. Severity of illness was measured by the ICNARC model [13]. The ICNARC model encompasses a weighting for acute physiology (the ICNARC physiology score, defined by derangement from the normal range for twelve physiological variables in the first 24 hours following admission to ICU) and additionally a weighting for age, diagnostic category coefficients and interactions with the physiology score, cardiopulmonary resuscitation within 24 hours prior to admission, and source of admission.

#### Treatment

Data were extracted on the proportion of people that had the following:

- lowest temperature of ≤ 34 °C in the first 24 h (as a surrogate marker for treatment with therapeutic hypothermia);
- treatment withdrawn;
- timing of treatment withdrawn.

#### Outcome

Survival data were extracted at discharge from the CMP unit and at ultimate discharge from an acute hospital. The proportion of survivors discharged to home was documented. In those who died, the proportion who became solid organ donors was collected.

#### Activity

Length of stay in the CMP unit was calculated in fractions of days from the dates and times of admission and discharge. Length of stay in hospital was calculated in days from the dates of original admission and ultimate discharge. Readmissions to the unit within the same hospital stay were identified from the postcode, date of birth and sex, and confirmed by the participating units.

### Statistical analyses

A statistical analysis plan was agreed a priori. The analyses performed were as follows.

#### Descriptive statistics

Case mix, withdrawal, outcome and activity were described annually for all admissions identified as post-cardiac arrest admissions, and separately for out-of-hospital cardiac arrest and in-hospital cardiac arrest. Readmissions were included in the descriptive statistics but were removed from the outcomes. To evaluate changes in study variables by calendar year we used logistic regression for categorical variables and linear regression for continuous, using the year of admission as the predictor variable. For non-normally distributed data, we used the Jonckheere-Terpstra trend test.

#### Multivariable analyses

A multivariable logistic regression was performed to analyse the impact on outcome of year of admission. To assess whether in-hospital mortality had improved over time, hierarchical multivariate logistic regression models were then constructed, with in-hospital mortality as the dependent variable, year of admission as the main exposure variable (modelled as continuous variable ranging from year 2004 to 2014), and ICU as a random effect. Models were adjusted for illness severity and potential confounding variables, including age, reason for admission and source for admission. This model estimated the adjusted probability of in-hospital mortality per incremental year over the study period. Separated models were developed for out-of-hospital CPR admissions and in-hospital CPR admissions.

All analyses were repeated using only the data from those ICUs contributing data throughout the study period.

All analyses were performed using Stata 13.0 (Stata Corporation, College Station, TX, USA).

## Results

During the period 1 January 2004 to 31 December 2014, 1,338,031 admissions to 286 ICUs in England, Wales and Northern Ireland were included in the CMPD. Of these, 63,417 (4.7 %) received CPR within the 24 hours before admission to the ICU and received ventilation on admission.

The number of contributing ICUs, total number of admissions, and number of cardiac arrests (out-of-hospital and in-hospital) by year is shown in Table 1. The number of ICUs contributing data to the CMPD increased steadily during the period of study; 116 ICUs contributed data throughout the study period and separate analyses confined to these ICUs are presented in Additional file 1: Table S1, Additional file 2: Table S2, Additional file 3: Table S3. While mechanically ventilated survivors of cardiac arrest decreased as a percentage of all critical care unit admissions (from 5.1 % in 2004 to 4.7 % in 2014, *p* < 0.001), they represented an increasing proportion of mechanically ventilated admissions (from 9.0 % in 2004 to 12.2 % in 2014, *p* < 0.001).

**Table 1:** A total of 1,338,031 admissions to 286 adult general critical care units in England, Wales and Northern Ireland from 1 January 2004 to 31 December 2014

Year	Number of units	All admissions		Cardiac arrest admissions		
			Out-of-hospital n (%)	In-hospital n (%)	All n (%)	% of ventilated admissions
2004	170	79,681	1582 (2.0)	2446 (3.1)	4028 (5.1)	9.0
2005	170	80,729	1592 (2.0)	2424 (3.0)	4016 (5.0)	9.0
2006	173	82,371	1643 (2.0)	2364 (2.9)	4007 (4.9)	9.2
2007	184	90,053	1938 (2.2)	2418 (2.7)	4356 (4.8)	9.8
2008	194	99,962	2384 (2.4)	2453 (2.5)	4837 (4.8)	10.2
2009	211	109,788	2519 (2.3)	2704 (2.5)	5223 (4.8)	10.6
2010	225	131,785	2926 (2.2)	3178 (2.4)	6104 (4.6)	10.7
2011	235	149,737	3334 (2.2)	3366 (2.2)	6700 (4.5)	11.0
2012	235	161,953	3632 (2.2)	3843 (2.4)	7475 (4.6)	11.5
2013	235	165,770	3924 (2.4)	4083 (2.5)	8007 (4.8)	11.8
2014	254	186,202	4147 (2.2)	4517 (2.4)	8664 (4.7)	12.2
Total	286	1,338,031	29,621 (2.2)	33,796 (2.5)	63,417 (4.7)	10.6

**Fig. 1:**
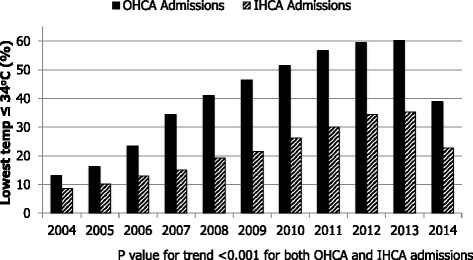
Proportion of post-cardiac arrest patients with lowest documented temperature ≤ 34 °C in the first 24 hours (2004–2014). *IHCA* in-hospital cardiac arrest, *OHCA* out of hospital cardiac arrest

The proportion of patients with a lowest temperature in the first 24 h of ≤ 34 °C for both out-of-hospital and in-hospital cardiac arrests has increased steadily, peaking in 2013 (*p* <0.001); the proportion in both cohorts decreases substantially in 2014 (Fig. 1, Tables 2 and 3).

**Table 2:** Trends in characteristics and mortality for ICU admissions following out-of-hospital cardiac arrest

	2004	2005	2006	2007	2008	2009	2010	2011	2012	2013	2014	*p* value for trend
Number of admissions after cardiac arrest, n (%)	1582 (2.0)	1592 (2.0)	1643 (2.0)	1938 (2.2)	2384 (2.4)	2519 (2.3)	2926 (2.2)	3334 (2.2)	3632 (2.2)	3924 (2.4)	4147 (2.2)	<0.001
Age, mean (SD)	60 (17.7)	61 (17.4)	61 (16.6)	60 (18.4)	60 (17.3)	60 (17.2)	61 (16.9)	61 (17.2)	61 (17.8)	61 (17.9)	61 (17.8)	0.478
Gender, males n (%)	954 (60.3)	1000 (62.8)	1022 (62.2)	1224 (63.2)	1529 (64.1)	1614 (64.1)	1878 (64.2)	2139 (64.2)	2382 (65.6)	2537 (64.7)	2626 (63.3)	0.006
ICNARC Physiology Score, mean (SD)	28 (10.0)	28 (10.1)	28 (10.3)	28 (10.0)	28 (10.0)	28 (9.9)	29 (9.9)	29 (9.6)	29 (9.9)	30 (10.0)	29 (10.5)	<0.001
Critical care unit length of stay, mean (SD)	4 (6.8)	4 (5.9)	4 (5.9)	5 (7.3)	4 (6.5)	5 (6.8)	5 (7.4)	5 (7.7)	5 (7.4)	5 (8.1)	5 (8.7)	<0.001^*^
Critical care unit length of stay, median (IQR)	1.9 (0.8 4.2)	2.0 (0.8 4.1)	2.1 (0.9 4.5)	2.3 (1.0 5.0)	2.3 (1.0 4.8)	2.6 (1.1 5.1)	2.6 (1.0 5.3)	2.8 (1.1 5.7)	2.8 (1.2 5.7)	2.9 (1.1 5.8)	2.7 (1.0 5.9)	<0.001^*^
Hospital length of stay, mean (SD)	13 (28.5)	14 (26.6)	14 (24.1)	14 (25.5)	14 (27.2)	15 (30.3)	14 (30.3)	14 (27.8)	13 (25.7)	13 (24.9)	14 (25.3)	0.0020^*^
Hospital length of stay, median (IQR)	3.0 (1.0 12.0)	3.5 (1.0 14.0)	4.0 (1.0 16.0)	4.0 (1.0 15.0)	4.0 (1.0 16.0)	5.0 (1.0 15.0)	4.0 (1.0 15.0)	5.0 (2.0 15.0)	5.0 (1.0 16.0)	4.0 (1.0 15.0)	5.0 (1.0 14.0)	0.0020^*^
lowest temperature of ≤ 34 °C 24 h n (%)	202 (13.1)	250 (16.2)	377 (23.5)	654 (34.5)	964 (41.0)	1160 (46.4)	1495 (51.5)	1870 (56.7)	2143 (59.5)	2344 (60.2)	1603 (38.9)	<0.001
Treatment withdrawn n (%)	498 (31.5)	483 (30.3)	512 (31.2)	573 (29.6)	753 (31.6)	807 (32.0)	972 (33.2)	1171 (35.1)	1240 (34.1)	1394 (35.6)	1444 (34.8)	<0.001
Time to treatment withdrawn (days) mean (SD)	3 (3.8)	3 (3.1)	4 (3.2)	4 (4.1)	4 (2.8)	4 (3.4)	4 (3.3)	4 (4.4)	4 (3.8)	5 (4.6)	4 (4.0)	<0.001^*^
Time to treatment withdrawn (days) median (IQR)	2.5 (1.7 3.9)	2.6 (1.9 3.7)	2.5 (1.9 4.1)	2.7 (1.8 4.2)	2.9 (2.0 4.3)	2.9 (1.9 4.5)	3.2 (2.0 4.7)	3.3 (2.1 5.2)	3.2 (2.1 5.0)	3.3 (2.1 5.5)	3.3 (2.0 5.4)	<0.001^*^
Solid organ donor n (%)^**^	34 (3.1)	22 (2.1)	33 (3.1)	50 (4.0)	77 (5.1)]	90 (5.6)	126 (6.6)	155 (7.4)	198 (8.7)	217 (8.4)	278 (10.1)	<0.001
ICU mortality, n (%)	919 (58.1)	856 (53.8)	859 (52.3)	1009 (52.1)	1248 (52.3)	1348 (53.5)	1620 (55.4)	1800 (54.0)	1957 (53.9)	2255 (57.5)	2361 (56.9)	<0.001
Hospital mortality, n (%)	1098 (70.1)	1059 (67.5)	1076 (66.3)	1251 (65.3)	1514 (64.4)	1617 (64.8)	1917 (65.9)	2089 (63.1)	2287 (63.2)	2590 (66.3)	2739 (66.4)	0.024
Survivors discharged home, n (%)^***^	354 (73.1)	375 (70.4)	383 (67.6)	440 (64.0)	550 (63.2)	575 (63.7)	654 (64.8)	817 (65.6)	850 (63.2)	842 (63.1)	881 (62.6)	<0.001

*ICU* intensive care unit, *ICNARC* Intensive Care National Audit & Research Centre, *IQR* interquartile range, *SD* standard deviation

^*^Jonckheere-Terpstra test; ^**^ percentage of hospital deaths, ^***^percentage of hospital survivors

**Table 3:** Trends in characteristics and mortality for ICU admissions following in-hospital cardiac arrest

	2004	2005	2006	2007	2008	2009	2010	2011	2012	2013	2014	*p* value for trend
Number of admissions after cardiac arrest, n (%)	2446 (3.1)	2424 (3.0)	2364 (2.9)	2418 (2.7)	2453 (2.5)	2704 (2.5)	3178 (2.4)	3366 (2.2)	3,843 (2.4)	4083 (2.5)	4517 (2.4)	<0.001
Age, mean (SD)	64 (16.3)	63 (16.7)	65 (15.6)	64 (16.3)	64 (16.5)	64 (16.2)	64 (15.8)	64 (15.6)	65 (15.3)	65 (15.2)	65 (15.3)	<0.001
Gender, males n (%)	1404 (57.4)	1400 (57.8)	1413 (59.8)	1439 (59.5)	1496 (61.0)	1623 (60.0)	1893 (59.6)	2059 (61.2)	2399 (62.4)	2554 (62.6)	2916 (64.6)	<0.001
ICNARC Physiology Score, mean (SD)	29 (11.2)	29 (10.9)	29 (11.1)	29 (10.9)	29 (10.6)	29 (10.5)	29 (10.4)	29 (10.2)	29 (10.1)	29 (10.4)	28 (10.5)	0.121
Critical care unit length of stay, mean (SD)	5 (9.0)	6 (10.1)	5 (8.5)	5 (9.5)	6 (10.1)	6 (9.9)	6 (10.4)	7 (12.2)	6 (9.4)	6 (9.4)	6 (10.5)	<0.001^*^
Critical care unit length of stay, median (IQR)	2.0 (0.6 5.8)	2.2 (0.7 6.3)	2.0 (0.6 5.9)	2.2 (0.8 6.0)	2.5 (0.8 7.1)	2.5 (0.9 6.9)	2.7 (0.9 6.7)	2.9 (1.0 7.1)	3.2 (1.1 7.4)	3.1 (1.0 7.3)	3.2 (1.1 7.4)	<0.001^*^
Hospital length of stay, mean (SD)	22 (35.8)	24 (39.7)	21 (32.3)	23 (34.2)	22 (33.0)	22 (37.5)	23 (43.9)	23 (37.4)	22 (33.1)	22 (35.9)	21 (33.4)	0.1128 ^*^
Hospital length of stay, median (IQR)	10.0 (3.0 27.0)	11.0 (3.0 28.0)	10.0 (3.0 26.0)	11.0 (3.0 26.0)	11.0 (4.0 28.0)	11.0 (3.0 26.0)	10.0 (3.0 26.0)	11.0 (4.0 27.0)	11.0 (4.0 27.0)	10.0 (4.0 26.0)	11.0 (4.0 25.0)	0.1128 ^*^
lowest temperature of ≤ 34 °C 24 h n (%)	200 (8.6)	237 (10.2)	293 (12.9)	352 (15.0)	464 (19.3)	575 (21.5)	817 (26.2)	997 (30.0)	1305 (34.5)	1,420 (35.3)	1014 (22.8)	<0.001
Treatment withdrawn n (%)	672 (27.5)	638 (26.3)	602 (25.5)	628 (26.0)	682 (27.8)	786 (29.1)	927 (29.2)	923 (27.4)	1149 (29.9)	1,198 (29.4)	1336 (29.6)	<0.001
Time to treatment withdrawn (days) mean (sd)	4 (6.6)	4 (5.2)	4 (5.6)	4 (5.4)	5 (6.5)	5 (7.2)	5 (8.8)	5 (7.3)	5 (6.5)	5 (6.9)	5 (6.1)	<0.001^*^
Time to treatment withdrawn (days) median (IQR)	2.4 (1.5 4.3)	2.4 (1.5 4.6)	2.4 (1.6 4.8)	2.5 (1.6 4.6)	2.6 (1.5 5.4)	2.7 (1.8 5.1)	2.9 (1.8 5.3)	3.2 (1.8 5.5)	3.3 (1.9 5.8)	3.2 (1.9 6.4)	3.4 (2.0 6.3)	<0.001^*^
Solid organ donor n (%)^**^	25 (1.5)	17 (1.1)	17 (1.1)	24 (1.6)	30 (1.9)	45 (2.6)	38 (1.9)	50 (2.5)	73 (3.2)	85 (3.5)]	104 (3.9)	<0.001
ICU mortality, n (%)	1406 (57.5)	1308 (54.0)	1302 (55.1)	1234 (51.0)	1281 (52.2)	1434 (53.1)	1662 (52.3)	1631 (48.5)	1909 (49.7)	2023 (49.6)	2235 (49.5)	<0.001
Hospital mortality, n (%)	1683 (70.4)	1592 (66.7)	1593 (68.8)	1526 (64.2)	1555 (64.6)	1719 (64.5)	1988 (63.4)	1965 (59.2)	2280 (60.0)	2400 (59.5)	2687 (60.3)	<0.001
Survivors discharged home, n (%)^***^	521 (68.3)	583 (70.1)	530 (68.7)	622 (69.7)	624 (69.5)	681 (69.1)	839 (70.5)	1012 (72.2)	1136 (72.7)	1209 (71.8)	1292 (70.6)	0.559

*ICU* intensive care unit, *ICNARC* Intensive Care National Audit & Research Centre, *IQR* interquartile range, *SD* standard deviation

^*^Jonckheere-Terpstra test; ^**^ percentage of hospital deaths; ^***^percentage of hospital survivors

**Fig. 2:**
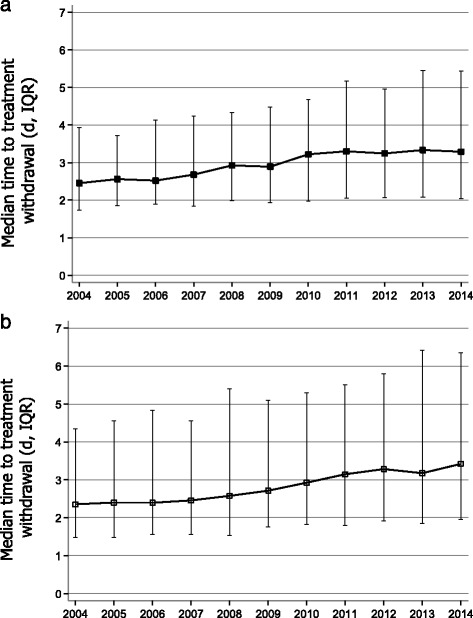
**a** Time to treatment withdrawal following out-of-hospital cardiac arrest 2004–2014 (median, IQR) (*p* < 0.001). **b** Time to treatment withdrawal following in-hospital cardiac arrest 2004–2014 (median, IQR) (*p* < 0.001). *IQR* interquartile range

During 2004–2014 the mean ICNARC physiology scores have increased slightly for out-of-hospital cardiac arrest but are unchanged for the in-hospital cohort. The proportion of post-cardiac arrest patients in whom treatment was withdrawn has increased over the analysis period: out-of-hospital cardiac arrest 31.5 % in 2004 versus 34.8 % in 2014 (*p* <0.001); in-hospital cardiac arrest 27.5 % in 2004 versus 29.6 % in 2014 (*p* <0.001). The time to treatment withdrawal has increased over the period of analysis (out-of-hospital cardiac arrest median [IQR] = 2.5 [1.7, 3.9] days in 2004 versus 3.3 [2.0, 5.4] days in 2014 (*p* <0.001); in-hospital cardiac arrest = 2.4 [1.5, 4.3] days in 2004 versus 3.4 [2.0, 6.3] in 2014 (*p* <0.001) (Fig. 2a and b).

**Fig. 3:**
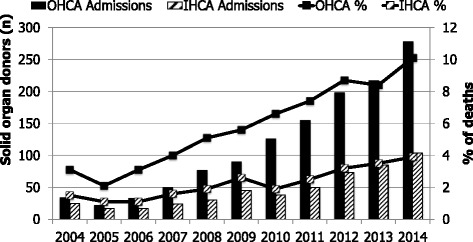
Solid organ donors among those dying after admission to an intensive care unit following cardiopulmonary resuscitation (2004–2014). *IHCA* in-hospital cardiac arrest, *OHCA* out of hospital cardiac arrest

During the analysis period there has been an increase in both the absolute number of solid organ donors and the proportion of those dying who become solid organ donors (Fig. 3). The increase is particular marked among those dying after out-of-hospital cardiac arrests.

The critical care length of stay (median [IQR]) has increased from 1.9 [0.8, 4.2] to 2.7 [1.0, 5.9] days (*p* value for a trend <0.001) following out-of-hospital cardiac arrest and from 2.0 [0.6, 5.8] to 3.2 [1.1, 7.4] (*p* value for a trend <0.001) following in-hospital cardiac arrest. Length of ICU and hospital stay for survivors and non-survivors is given in the Additional file 4: Table S4 and Additional file 5: Table S5. Length of ICU stay for both survivors and non-survivors has increased significantly; hospital length of stay is unchanged following out-of-hospital cardiac arrest but is significantly shorter after in-hospital cardiac arrest.

**Fig. 4:**
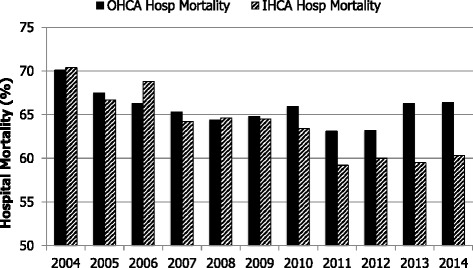
Ultimate acute hospital mortality (2004–2014). *IHCA* in-hospital cardiac arrest, *OHCA* out of hospital cardiac arrest

Over the period of analysis, the ultimate acute hospital mortality for out-of-hospital cardiac arrest has decreased from 70.1 % to 66.4 % (*p* value for trend 0.024) and for in-hospital cardiac arrest it has decreased from 70.4 % to 60.3 % (*p* value for trend <0.001) cohorts (Fig. 4). After adjustment for case mix, the reduction in hospital mortality remained significant following both out-of-hospital CPR (adjusted odds ratio (OR) per year 0.96 (0.95 to 0.97); *p* value for trend <0.001) and in-hospital CPR (adjusted OR per year 0.96 (0.95 to 0.97); *p* value for trend <0.001). The critical care unit mortality for out-of-hospital cardiac arrests has decreased from 58.1 % to 56.9 % (*p* value for trend <0.001) and for in-hospital cardiac arrest it has decreased from 57.5 % to 49.5 % (*p* value for trend <0.001). The proportion of out-of-hospital cardiac arrest survivors discharged home has decreased from 73.1 % to 62.6 % (*p* value for a trend <0.001). In contrast, the proportion of in-hospital cardiac arrest survivors discharged home has remained unchanged at about 70 % throughout 2004–2014 (*p* value for a trend 0.559). During 2004–2014 the discharge destinations of survivors following out-of-hospital cardiac arrest were home (66.3 %); other hospital (22.4 %) and rehabilitation (7.3 %). The discharge destinations of survivors of in-hospital cardiac arrest were home (73.6 %); other hospital (16.5 %) and rehabilitation (7.4 %).

Re-analysis using only the 116 ICUs contributing data throughout the study period produced results consistent with the primary analysis of all ICUs (Additional file 1: Table S1, Additional file 2: Table S2, Additional file 3: Table S3). The adjusted OR per year for the reduction in hospital mortality following both out-of-hospital and in-hospital cardiac arrest are 0.96 (0.95 to 0.97; *p* value for trend <0.001).

## Discussion

We have documented a substantial increase in the proportion of mechanically ventilated patients admitted to ICU who have undergone cardiopulmonary resuscitation before admission (9.0 % in 2004 and 12.2 % 2014); the largest increase is in the proportion of out-of-hospital cardiac arrests (3.5 % in 2004 and 5.8 % in 2014).

There has been a reduction in the risk-adjusted hospital mortality following admission to ICU after both out-of-hospital and in-hospital cardiac arrest (OR 0.96 per year) and re-analysis of only the 116 ICUs contributing data throughout the study period shows the same results. This finding is consistent with reports of increasing survival after admission to ICUs in Finland and the Netherlands [8, 9]. We have documented a significant reduction in the proportion of survivors who are discharged home after admission to ICU after out-of-hospital cardiac arrest. Neurological status on discharge is not included in the CMP database but the reduction in the proportion of survivors being discharged home after out-of-hospital cardiac arrest might indicate that the neurological outcome for these individuals is slightly worse over the period of study. This trend is not a feature among the survivors of in-hospital cardiac arrest. We do not know if discharge to home is a reliable surrogate for neurological outcome and this requires further study.

Trends of reducing ICU mortality have also been shown for other pathologies and relative to some of these (e.g. severe sepsis) the reduction in mortality that we have shown for cardiac arrest patients is comparatively modest [14].

Like the previous studies from Finland and the Netherlands, we have also shown a substantial increase in the implementation of mild induced hypothermia (increasing from 13.1 % in 2004 and peaking at 38.9 % in 2013 in the out-of-hospital cohort). In 2014 there was a substantial decrease in the proportion of patients (in both groups) with a documented lowest temperature in the first 24 hours. The results of a randomised trial of targeted temperature management (TTM) at 33 °C versus 36 °C after cardiac arrest were published in 2013 [15]. The TTM trial showed no difference in all-cause mortality between the two groups. Our findings may reflect a change in practice as a result of this study, with clinicians electing to use a target temperature of 36 °C instead of 33 °C or possibly abandoning the use of TTM altogether. There have been several other developments (e.g. changes in prognostication strategy, and use of percutaneous coronary intervention) in post-resuscitation care during the study period and one or more of these could contribute to improved survival.

We used the ICNARC model instead of Acute Physiology and Chronic Health Evaluation (APACHE) II for risk adjustment because we have shown previously that the ICNARC model gives much better discrimination than APACHE II in post-cardiac arrest patients [1]. In APACHE II, CPR is treated as a diagnostic category and is given the weighting for outcome prediction, overriding any other diagnostic category. In the ICNARC model, CPR receives a weighting but this is in addition to a separate diagnostic category weighting based on the primary reason for admission.

Our data document a significant change in the ICU management of post-cardiac arrest patients. The proportion of patients in whom treatment was withdrawn has increased slightly after both out-of-hospital and in-hospital cardiac arrest but the median time to treatment withdrawal has increased considerably from 2.5 to 3.3 days for out-of-hospital cardiac arrests and from 2.4 to 3.4 days after in-hospital cardiac arrest. For the last few years many investigators have advocated a much more cautious approach to prognostication, including delaying decisions to withdraw life-sustaining treatment until at least 3 days after cardiac arrest [16–19]. Our data suggest that in this respect practice is already changing [20].

Post-cardiac arrest patients who die after ICU admission are increasingly being recognised as potential solid organ donors [21, 22]. Organ survival rates after donation from post-cardiac arrest patients are comparable to those from other donors [21, 23]. Over the 11-year period of our study there has been a substantial increase in the proportion of non-survivors who have become solid organ donors: for out-of-hospital cardiac arrests this proportion has increased from 3.1 % (34) of non-survivors in 2004 to 10.1 % (278) in 2014. The latest National Health Service Blood and Transplant activity report (http://www.odt.nhs.uk/uk-transplant-registry/annual-activity-report/) indicates that, excluding Scotland (Scottish ICUs do not contribute data to the ICNARC CMP), there were 1185 deceased donors in the UK for the 2014–2015 year. Given that our data does not include every ICU in England, Wales and Northern Ireland, it seems likely that those admitted to an ICU after out-of-hospital cardiac arrest account for at least 25 % of the deceased solid organ donors in the UK.

Our study has several weaknesses. The CMP was not designed to collect data on cardiac arrest patients specifically and many of the essential data included in the Utstein template for reporting cardiac arrest data are missing [24]. Most notable among these are the presumed aetiology of the cardiac arrest, first monitored rhythm, bystander CPR and neurological outcome. The CMP dataset was modified to include location of cardiac arrest in 2009 but as this was not recorded throughout our study period, we have inferred this from the location of the patient before admission to the ICU and applied this to all patients in the study. We made inferences about the location of cardiac arrest: patients having a cardiac arrest in the emergency department will have been included in the out-of-hospital group. We have used lowest temperature within the first 24 h of ≤ 34 °C as a surrogate for the use of mild induced hypothermia but it is possible that some of these patients spontaneously had a temperature of less than 34 °C. By including only those patients undergoing mechanical ventilation at the time of admission to the ICU, we have attempted to exclude those patients who had only a brief period of CPR within 24 hours of admission, but required admission to the ICU for other reasons. We have documented the proportion of hospital survivors who were discharged home but this has not been validated as a surrogate for good neurological outcome.

## Conclusions

In conclusion, during the period 2004–2014 survivors of cardiac arrest accounted for an increasing proportion of mechanically ventilated admissions to ICUs in the ICNARC CMP (9.0 % in 2004 increasing to 12.2 % in 2014). Risk-adjusted hospital mortality following admission to ICU after cardiac arrest has decreased significantly during this period (OR 0.96 per year). Over this time, the ICU length of stay and time to treatment withdrawal has increased significantly. It seems likely that non-survivors of out-of-hospital cardiac arrest account for at least a quarter of the solid organ donors in the UK.

## Abbreviations

APACHE, Acute Physiology and Chronic Health Evaluation; CMPD, Case Mix Programme Database; CPR, cardiopulmonary resuscitation; ICNARC, Intensive Care National Audit & Research Centre; ICU, intensive care unit; IHCA, in-hospital cardiac arrest; IQR, interquartile range; OHCA, out-of-hospital cardiac arrest; TTM, targeted temperature management

## Additional files

Additional file 1: Table S1. A total of 753,290 admissions to the 116 adult general critical care units in England, Wales and Northern Ireland that contributed data throughout the period 1 January 2004 to 31 December 2014. (DOCX 74 kb) Additional file 2: Table S2. Trends in characteristics and mortality for ICU admissions following out-of-hospital cardiac arrest. Data from the 116 ICUs contributing data throughout the study period. (DOCX 16 kb) Additional file 3: Table S3. Trends in characteristics and mortality for ICU admissions following in-hospital cardiac arrest. Data from the 116 ICUs contributing data throughout the study period. (DOCX 16 kb) Additional file 4: Table S4. Length of stay for survivors and non-survivors. Data from all 286 ICUs contributing data to the case mix programme database. (DOCX 14 kb) Additional file 5: Table S5. Length of stay for survivors and non-survivors. Data from the 116 ICUs contributing data throughout the study period. (DOCX 90 kb)

## References

[1] Nolan JP, Laver SR, Welch CA, Harrison DA, Gupta V, Rowan K (2007). Outcome following admission to UK intensive care units after cardiac arrest: a secondary analysis of the ICNARC Case Mix Programme Database. Anaesthesia..

[2] Nolan JP, Soar J, Cariou A (2015). European Resuscitation Council and European Society of Intensive Care Medicine 2015 guidelines for post-resuscitation care. Intensive Care Med..

[3] Nolan JP, Soar J, Cariou A (2015). European Resuscitation Council and European Society of Intensive Care Medicine guidelines for post-resuscitation care 2015: Section 5 of the European Resuscitation Council Guidelines for Resuscitation. Resuscitation..

[4] Sasson C, Rogers MA, Dahl J, Kellermann AL (2010). Predictors of survival from out-of-hospital cardiac arrest: a systematic review and meta-analysis. Circ Cardiovasc Qual Outcomes..

[5] Girotra S, Nallamothu BK, Spertus JA (2012). Trends in survival after in-hospital cardiac arrest. N Engl J Med..

[6] Chan PS, McNally B, Tang F, Kellermann A, Group CS (2014). Recent trends in survival from out-of-hospital cardiac arrest in the United States. Circulation..

[7] Fugate JE, Brinjikji W, Mandrekar JN (2012). Post-cardiac arrest mortality is declining: a study of the US National Inpatient Sample 2001 to 2009. Circulation..

[8] Reinikainen M, Oksanen T, Leppanen P (2012). Mortality in out-of-hospital cardiac arrest patients has decreased in the era of therapeutic hypothermia. Acta Anaesthesiol Scand..

[9] van der Wal G, Brinkman S, Bisschops LL (2011). Influence of mild therapeutic hypothermia after cardiac arrest on hospital mortality. Crit Care Med..

[10] Carr BG, Kahn JM, Merchant RM, Kramer AA, Neumar RW (2009). Inter-hospital variability in post-cardiac arrest mortality. Resuscitation..

[11] Keenan SP, Dodek P, Martin C, Priestap F, Norena M, Wong H (2007). Variation in length of intensive care unit stay after cardiac arrest: where you are is as important as who you are. Crit Care Med..

[12] Harrison DA, Brady AR, Rowan K (2004). Case mix, outcome and length of stay for admissions to adult, general critical care units in England, Wales and Northern Ireland: the Intensive Care National Audit & Research Centre Case Mix Programme Database. Crit Care..

[13] Harrison DA, Parry GJ, Carpenter JR, Short A, Rowan K (2007). A new risk prediction model for critical care: the Intensive Care National Audit & Research Centre (ICNARC) model. Crit Care Med..

[14] Kaukonen KM, Bailey M, Suzuki S, Pilcher D, Bellomo R (2014). Mortality related to severe sepsis and septic shock among critically ill patients in Australia and New Zealand, 2000-2012. JAMA..

[15] Nielsen N, Wetterslev J, Cronberg T (2013). Targeted temperature management at 33 degrees C versus 36 degrees C after cardiac arrest. N Engl J Med..

[16] Perman SM, Kirkpatrick JN, Reitsma AM (2012). Timing of neuroprognostication in postcardiac arrest therapeutic hypothermia. Crit Care Med..

[17] Grossestreuer AV, Abella BS, Leary M (2013). Time to awakening and neurologic outcome in therapeutic hypothermia-treated cardiac arrest patients. Resuscitation..

[18] Sandroni C, Cavallaro F, Callaway CW (2013). Predictors of poor neurological outcome in adult comatose survivors of cardiac arrest: a systematic review and meta-analysis. Part 2: Patients treated with therapeutic hypothermia. Resuscitation..

[19] Sandroni C, Cariou A, Cavallaro F (2014). Prognostication in comatose survivors of cardiac arrest: an advisory statement from the European Resuscitation Council and the European Society of Intensive Care Medicine. Resuscitation..

[20] Binks AC, Murphy RE, Prout RE (2010). Therapeutic hypothermia after cardiac arrest - implementation in UK intensive care units. Anaesthesia..

[21] Sandroni C, Adrie C, Cavallaro F (2010). Are patients brain-dead after successful resuscitation from cardiac arrest suitable as organ donors? A systematic review. Resuscitation..

[22] Reynolds JC, Rittenberger JC, Callaway CW, Post Cardiac Arrest Service (2014). Patterns of organ donation among resuscitated patients at a regional cardiac arrest center. Resuscitation..

[23] Soar J, Callaway CW, Aibiki M (2015). Part 4: Advanced life support: 2015 International Consensus on Cardiopulmonary Resuscitation and Emergency Cardiovascular Care Science With Treatment Recommendations. Resuscitation..

[24] Perkins GD, Jacobs IG, Nadkarni VM (2015). Cardiac arrest and cardiopulmonary resuscitation outcome reports: update of the Utstein Resuscitation Registry Templates for Out-of-Hospital Cardiac Arrest: a statement for healthcare professionals from a task force of the International Liaison Committee on Resuscitation (American Heart Association, European Resuscitation Council, Australian and New Zealand Council on Resuscitation, Heart and Stroke Foundation of Canada, InterAmerican Heart Foundation, Resuscitation Council of Southern Africa, Resuscitation Council of Asia); and the American Heart Association Emergency Cardiovascular Care Committee and the Council on Cardiopulmonary, Critical Care, Perioperative and Resuscitation. Resuscitation.

