# Supplementary material for: Increasing survival after admission to UK critical care units following cardiopulmonary resuscitation
Source: Crit Care. 2016 Jul 9;20:219. doi: 10.1186/s13054-016-1390-6 (PMC4938902; doi:10.1186/s13054-016-1390-6)
Supplement: Additional file 1: Table S1. — A total of 753,290 admissions to the 116 adult general critical care units in England, Wales and Northern Ireland that contributed data throughout the period 1 January 2004 to 31 December 2014. (DOCX 74 kb) [file 13054_2016_1390_MOESM1_ESM.docx]

Table S1. 753,290 admissions to the 116 adult general critical care units in England, Wales and Northern Ireland that contributed data throughout the period 1^st^ January 2004 to 31^st^ December 2014.

| **Year** | **All admissions** |  | **Cardiac arrest admissions** | | |
| --- | --- | --- | --- | --- | --- |
|  |  | **Out-of-hospital**  **n (%)** | **In-hospital**  **n (%)** | **All**  **n (%)** | **% of ventilated admissions** |
| 2004 | 56,899 | 1,582 (2.0) | 1,186 (2.1) | 1,759 (3.1) | 9.3 |
| 2005 | 58,437 | 1,592 (2.0) | 1,236 (2.1) | 1,833 (3.1) | 9.6 |
| 2006 | 59,130 | 1,643 (2.0) | 1,207 (2.0) | 1,813 (3.1) | 9.5 |
| 2007 | 60,512 | 1,938 (2.2) | 1,434 (2.4) | 1,734 (2.9) | 10.4 |
| 2008 | 63,122 | 2,384 (2.4) | 1,649 (2.6) | 1,629 (2.6) | 10.8 |
| 2009 | 64,169 | 2,519 (2.3) | 1,629 (2.5) | 1,712 (2.7) | 11.3 |
| 2010 | 70,984 | 2,926 (2.2) | 1,752 (2.5) | 1,855 (2.6) | 11.3 |
| 2011 | 73,602 | 3,334 (2.2) | 1,914 (2.6) | 1,839 (2.5) | 11.7 |
| 2012 | 79,656 | 3,632 (2.2) | 2,083 (2.6) | 2,132 (2.7) | 12.5 |
| 2013 | 81,938 | 3,924 (2.4) | 2,282 (2.8) | 2,193 (2.7) | 13.0 |
| 2014 | 84,841 | 4,147 (2.2) | 2,271 (2.7) | 2,340 (2.8) | 13.2 |
| **Total** | 753,290 | 29,621 (2.2) | 18,643 (2.5) | 20,839 (2.8) | 11.2 |
