# Supplementary material for: Increasing survival after admission to UK critical care units following cardiopulmonary resuscitation
Source: Crit Care. 2016 Jul 9;20:219. doi: 10.1186/s13054-016-1390-6 (PMC4938902; doi:10.1186/s13054-016-1390-6)
Supplement: Additional file 2: Table S2. — Trends in characteristics and mortality for ICU admissions following out-of-hospital cardiac arrest. Data from the 116 ICUs contributing data throughout the study period. (DOCX 16 kb) [file 13054_2016_1390_MOESM2_ESM.docx]

Table S2. Trends in characteristics and mortality for ICU admissions following out-of-hospital cardiac arrest. Data from the 116 ICUs contributing data throughout the study period.

|  | 2004 | 2005 | 2006 | 2007 | 2008 | 2009 | 2010 | 2011 | 2012 | 2013 | 2014 | p value for trend |
| --- | --- | --- | --- | --- | --- | --- | --- | --- | --- | --- | --- | --- |
| Number of admissions after cardiac arrest, n (%) | 1,186 (2.1) | 1,236 (2.1) | 1,207 (2.0) | 1,434 (2.4) | 1,649 (2.6) | 1,629 (2.5) | 1,752 (2.5) | 1,914 (2.6) | 2,083 (2.6) | 2,282 (2.8) | 2,271 (2.7) | <0.001 |
| Age, mean (sd) | 60 (17.6) | 62 (17.4) | 61 (16.8) | 59 (18.6) | 60 (17.6) | 60 (17.3) | 61 (17.2) | 61 (17.2) | 61 (17.4) | 60 (17.6) | 60 (17.7) | 0.809 |
| Gender, males n (%) | 708 (60.1) | 768 (62.3) | 753 (62.6) | 906 (63.1) | 1,050 (64.3) | 1,034 (64.0) | 1,119 (64.9) | 1,207 (63.8) | 1,357 (65.4) | 1,463 (64.8) | 1,399 (63.1) | 0.015 |
| ICNARC Physiology Score, mean (sd) | 28 (10.1) | 28 (10.1) | 28 (10.1) | 28 (10.0) | 28 (10.0) | 28 (9.9) | 28 (9.5) | 29 (9.6) | 29 (9.5) | 29 (9.7) | 29 (10.4) | <0.001 |
| Critical care unit length of stay, mean (sd) | 4 (6.5) | 4 (5.9) | 4 (5.7) | 5 (7.0) | 4 (6.2) | 5 (6.3) | 5 (7.1) | 5 (7.9) | 5 (8.2) | 5 (7.8) | 5 (8.4) | <0.001* |
| Critical care unit length of stay, median (IQR) | 1.9 (0.8 4.1) | 1.9 (0.8 3.9) | 2.1 (0.9 4.5) | 2.3 (1.0 4.8) | 2.3 (1.0 4.7) | 2.6 (1.1 4.9) | 2.6 (1.1 5.2) | 2.8 (1.2 5.8) | 2.8 (1.2 5.5) | 3.0 (1.2 5.9) | 2.7 (1.0 5.9) | <0.001* |
| Hospital length of stay, mean (sd) | 12 (27.1) | 14 (26.6) | 14 (24.0) | 14 (24.2) | 14 (27.9) | 15 (30.4) | 14 (30.3) | 13 (25.4) | 13 (22.8) | 13 (23.1) | 13 (25.2) | <0.001* |
| Hospital length of stay, median (IQR) | 3.0 (1.0 12.0) | 4.0 (1.0 15.0) | 4.0 (1.0 16.0) | 4.0 (1.0 16.0) | 4.0 (1.0 16.0) | 5.0 (1.0 15.0) | 4.0 (1.0 15.0) | 5.0 (2.0 15.0) | 5.0 (2.0 16.5) | 5.0 (1.0 14.5) | 5.0 (1.0 14.0) | <0.001* |
| lowest temperature of ≤ 34 ^o^C 24 h n (%) | 153 (13.3) | 213 (17.8) | 292 (24.8) | 511 (36.5) | 689 (42.8) | 770 (48.1) | 894 (52.4) | 1,052 (56.4) | 1,248 (60.7) | 1,374 (61.4) | 842 (38.4) | <0.001 |
| Treatment withdrawn n (%) | 375 (31.8) | 378 (30.7) | 383 (31.9) | 426 (29.7) | 522 (32.0) | 527 (32.6) | 589 (34.2) | 689 (36.4) | 715 (34.5) | 821 (36.4) | 774 (34.9) | <0.001 |
| Time to treatment withdrawn (days) mean (sd) | 3 (3.2) | 3 (3.3) | 4 (3.3) | 4 (3.6) | 3 (2.8) | 4 (3.3) | 4 (2.8) | 4 (5.0) | 4 (3.3) | 5 (4.2) | 4 (3.7) | <0.001* |
| Time to treatment withdrawn (days) median (IQR) | 2.4 (1.7 4.1) | 2.5 (1.9 3.6) | 2.5 (1.9 4.1) | 2.6 (1.8 4.2) | 2.9 (1.9 4.3) | 2.9 (2.0 4.5) | 3.3 (2.0 4.8) | 3.4 (2.1 5.3) | 3.2 (2.0 4.8) | 3.4 (2.1 5.6) | 3.3 (2.0 5.4) | <0.001* |
| Solid organ donor n (%)** | 24 (2.9) | 18 (2.2) | 27 (3.5) | 35 (3.8) | 47 (4.6) | 58 (5.7) | 75 (6.6) | 97 (8.1) | 124 (9.7) | 123 (8.3) | 146 (10.1) | <0.001 |
|  |  |  |  |  |  |  |  |  |  |  |  |  |
| ICU mortality, n (%) | 692 (58.7) | 654 (53.0) | 619 (51.5) | 740 (51.5) | 844 (51.7) | 852 (52.7) | 945 (54.8) | 1,034 (54.7) | 1,105 (53.3) | 1,288 (57.1) | 1,252 (56.5) | 0.024 |
| Hospital Mortality, n (%) | 822 (70.5) | 816 (67.0) | 782 (65.7) | 922 (64.9) | 1,030 (63.7) | 1,021 (63.7) | 1,129 (65.9) | 1,202 (64.0) | 1,284 (62.1) | 1,487 (66.1) | 1,452 (65.9) | 0.024 |
| Survivors discharged home,  n (%)*** | 261 (75.9) | 298 (74.1) | 289 (70.8) | 330 (66.1) | 392 (66.9) | 391 (67.2) | 387 (66.2) | 457 (67.7) | 499 (63.8) | 503 (65.9) | 483 (64.1) | <0.001 |

* Jonckheere-Terpstra test ** percentage of hospital deaths ***percentage of hospital survivors
