# Supplementary material for: Increasing survival after admission to UK critical care units following cardiopulmonary resuscitation
Source: Crit Care. 2016 Jul 9;20:219. doi: 10.1186/s13054-016-1390-6 (PMC4938902; doi:10.1186/s13054-016-1390-6)
Supplement: Additional file 3: Table S3. — Trends in characteristics and mortality for ICU admissions following in-hospital cardiac arrest. Data from the 116 ICUs contributing data throughout the study period. (DOCX 16 kb) [file 13054_2016_1390_MOESM3_ESM.docx]

Table S3. Trends in characteristics and mortality for ICU admissions following in-hospital cardiac arrest. Data from the 116 ICUs contributing data throughout the study period.

|  | 2004 | 2005 | 2006 | 2007 | 2008 | 2009 | 2010 | 2011 | 2012 | 2013 | 2014 | p value for trend |
| --- | --- | --- | --- | --- | --- | --- | --- | --- | --- | --- | --- | --- |
| Number of admissions after cardiac arrest, n (%) | 1,759 (3.1) | 1,833 (3.1) | 1,813 (3.1) | 1,734 (2.9) | 1,629 (2.6) | 1,712 (2.7) | 1,855 (2.6) | 1,839 (2.5) | 2,132 (2.7) | 2,193 (2.7) | 2,340 (2.8) | <0.001 |
| Age, mean (sd) | 64 (16.2) | 63 (16.7) | 65 (15.8) | 64 (16.4) | 63 (16.5) | 64 (16.1) | 64 (16.2) | 64 (15.6) | 65 (15.2) | 65 (15.6) | 65 (15.1) | <0.001 |
| Gender, males n (%) | 1,003 (57.2) | 1,052 (57.4) | 1,092 (60.6) | 1,029 (58.9) | 1,018 (61.1) | 1,038 (60.1) | 1,125 (60.1) | 1,093 (59.8) | 1,321 (62.2) | 1,357 (62.4) | 1,471 (64.0) | <0.001 |
| ICNARC Physiology Score, mean (sd) | 29 (11.3) | 29 (10.8) | 30 (11.0) | 29 (10.9) | 29 (10.6) | 29 (10.4) | 29 (10.3) | 29 (10.1) | 29 (9.7) | 29 (10.2) | 28 (10.2) | 0.001 |
| Critical care unit length of stay, mean (sd) | 5 (8.7) | 6 (10.0) | 5 (8.5) | 5 (9.4) | 6 (9.2) | 6 (10.2) | 6 (9.7) | 6 (12.5) | 6 (9.0) | 6 (9.5) | 6 (8.7) | <0.001* |
| Critical care unit length of stay, median (IQR) | 1.9 (0.6 5.6) | 2.1 (0.7 6.1) | 1.9 (0.6 5.5) | 2.1 (0.8 5.9) | 2.5 (0.8 7.0) | 2.7 (0.8 7.0) | 2.6 (0.9 6.2) | 2.9 (1.0 6.7) | 3.3 (1.1 7.4) | 3.0 (1.0 7.0) | 3.1 (1.2 6.9) | <0.001* |
| Hospital length of stay, mean (sd) | 22 (36.1) | 23 (38.3) | 20 (33.0) | 22 (33.7) | 23 (33.8) | 21 (32.7) | 22 (44.7) | 22 (35.8) | 22 (31.9) | 21 (33.5) | 20 (30.9) | 0.2309* |
| Hospital length of stay, median (IQR) | 11.0 (3.0 27.0) | 12.0 (4.0 28.0) | 10.0 (3.0 26.0) | 11.5 (4.0 27.0) | 12.0 (4.0 28.0) | 11.0 (4.0 27.0) | 10.0 (4.0 25.0) | 11.0 (4.0 27.0) | 12.0 (5.0 28.0) | 10.0 (4.0 25.0) | 10.0 (4.0 24.0) | 0.2309* |
| lowest temperature of ≤ 34 ^o^C 24 h n (%) | 151 (9.0) | 186 (10.7) | 245 (14.2) | 262 (15.5) | 326 (20.1) | 368 (21.7) | 486 (26.6) | 542 (30.2) | 737 (35.3) | 770 (35.8) | 499 (22.0) | <0.001 |
| Treatment withdrawn n (%) | 504 (28.7) | 513 (28.0) | 482 (26.7) | 471 (27.0) | 477 (28.6) | 543 (31.4) | 582 (31.1) | 551 (30.1) | 614 (28.9) | 672 (30.9) | 708 (30.8) | 0.001 |
| Time to treatment withdrawn (days) mean (sd) | 4 (6.5) | 4 (5.3) | 4 (5.5) | 4 (5.6) | 5 (6.7) | 5 (8.1) | 5 (6.4) | 5 (6.2) | 5 (7.3) | 5 (5.7) | 5 (5.6) | <0.001* |
| Time to treatment withdrawn (days) median (IQR) | 2.3 (1.4 4.3) | 2.4 (1.4 4.4) | 2.4 (1.6 4.5) | 2.4 (1.5 4.3) | 2.5 (1.5 5.4) | 2.8 (1.7 5.3) | 2.9 (1.7 5.2) | 3.3 (1.9 5.5) | 3.5 (1.9 6.1) | 3.1 (1.8 5.7) | 3.4 (2.0 6.1) | <0.001* |
| Solid organ donor n (%)** | 20 (1.6) | 15 (1.2) | 15 (1.2) | 16 (1.4) | 15 (1.4) | 29 (2.6) | 22 (1.9) | 28 (2.6) | 44 (3.5) | 56 (4.3) | 60 (4.3) | <0.001 |
|  |  |  |  |  |  |  |  |  |  |  |  |  |
| ICU mortality, n (%) | 1,025 (58.4) | 1,012 (55.2) | 1,012 (56.2) | 903 (51.7) | 873 (52.4) | 928 (53.8) | 981 (52.4) | 920 (50.3) | 1,033 (48.6) | 1,087 (50.0) | 1,150 (50.0) | <0.001 |
| Hospital mortality, n (%) | 1,218 (70.6) | 1,224 (67.7) | 1,239 (69.6) | 1,108 (64.6) | 1,050 (64.1) | 1,108 (65.0) | 1,186 (64.2) | 1,089 (60.5) | 1,241 (59.0) | 1,309 (60.7) | 1,385 (61.1) | <0.001 |
| Survivors discharged home, n (%)*** | 370 (73.0) | 426 (72.9) | 400 (73.8) | 446 (73.4) | 437 (74.3) | 435 (72.9) | 476 (72.0) | 524 (73.6) | 646 (74.9) | 629 (74.3) | 649 (73.8) | 0.518 |

* Jonckheere-Terpstra test ** percentage of hospital deaths ***percentage of hospital survivors
