# Supplementary material for: Increasing survival after admission to UK critical care units following cardiopulmonary resuscitation
Source: Crit Care. 2016 Jul 9;20:219. doi: 10.1186/s13054-016-1390-6 (PMC4938902; doi:10.1186/s13054-016-1390-6)
Supplement: Additional file 4: Table S4. — Length of stay for survivors and non-survivors. Data from all 286 ICUs contributing data to the case mix programme database. (DOCX 14 kb) [file 13054_2016_1390_MOESM4_ESM.docx]

Table S4. Length of stay for survivors and non-survivors. Data from all 286 ICUs contributing data to the case mix programme database.

| Length of stay, days, median (IQR) | 2004 | 2005 | 2006 | 2007 | 2008 | 2009 | 2010 | 2011 | 2012 | 2013 | 2014 | p value for trend |
| --- | --- | --- | --- | --- | --- | --- | --- | --- | --- | --- | --- | --- |
| **Out-of-hospital cardiac arrest** |  |  |  |  |  |  |  |  |  |  |  |  |
| Survivors ICU stay | 3.0 (1.5 7.9) | 2.9 (1.4 6.5) | 3.2 (1.7 6.9) | 3.6 (1.9 8.8) | 3.3 (1.9 8.5) | 3.7 (2.0 8.8) | 4.0 (2.2 8.9) | 4.1 (2.3 9.3) | 4.5 (2.5 10.0) | 4.6 (2.8 9.5) | 4.7 (2.3 10.0) | <0.001 |
| Survivors hospital stay | 20.0 (10.0 38.0) | 19.0 (10.0 41.0) | 21.0 (11.0 38.0) | 21.0 (11.0 39.0) | 20.0 (11.0 37.0) | 20.0 (11.0 37.0) | 20.0 (12.0 36.0) | 19.0 (11.0 35.0) | 20.0 (11.0 36.0) | 20.0 (11.0 37.0) | 19.0 (11.0 36.0) | 0.341 |
| Non-survivors ICU stay | 1.5 (0.6 3.4) | 1.6 (0.6 3.2) | 1.6 (0.6 3.5) | 1.7 (0.7 3.8) | 1.8 (0.8 3.7) | 1.9 (0.8 3.9) | 1.9 (0.7 4.0) | 2.0 (0.7 4.1) | 2.0 (0.8 4.1) | 2.0 (0.7 4.4) | 2.0 (0.7 4.5) | <0.001 |
| Non-survivors hospital stay | 2.0 (1.0 4.0) | 2.0 (1.0 4.0) | 2.0 (1.0 5.0) | 2.0 (1.0 5.0) | 2.0 (1.0 4.0) | 2.0 (1.0 5.0) | 2.0 (1.0 5.0) | 2.0 (1.0 5.0) | 2.0 (1.0 5.0) | 2.0 (1.0 5.0) | 2.0 (1.0 5.0) | 0.001 |
|  |  |  |  |  |  |  |  |  |  |  |  |  |
| **In-hospital cardiac arrest** |  |  |  |  |  |  |  |  |  |  |  |  |
| Survivors ICU stay | 3.5 (1.8 9.8) | 4.3 (1.8 10.7) | 3.8 (1.8 9.3) | 3.6 (1.8 9.5) | 4.6 (2.0 11.9) | 4.5 (2.0 11.2) | 4.7 (2.3 10.6) | 4.5 (2.3 10.3) | 5.0 (2.6 11.2) | 5.0 (2.7 10.8) | 4.7 (2.3 10.3) | <0.001 |
| Survivors hospital stay | 27.0 (13.0 55.0) | 28.0 (15.0 54.0) | 26.0 (14.0 47.0) | 26.0 (13.0 50.0) | 27.0 (14.0 52.0) | 26.0 (14.0 50.0) | 24.0 (13.0 48.0) | 23.0 (12.0 45.0) | 23.0 (13.0 44.0) | 23.0 (12.0 45.0) | 22.0 (11.0 44.0) | <0.001 |
| Non-survivors ICU stay | 1.3 (0.4 4.2) | 1.5 (0.4 4.7) | 1.3 (0.3 4.2) | 1.5 (0.5 4.1) | 1.5 (0.4 4.9) | 1.7 (0.5 5.0) | 1.7 (0.4 4.7) | 1.9 (0.5 5.0) | 2.0 (0.6 5.3) | 1.9 (0.5 5.2) | 2.2 (0.6 5.6) | <0.001 |
| Non-survivors hospital stay | 6.0 (2.0 15.0) | 6.0 (2.0 15.0) | 5.0 (2.0 14.0) | 6.0 (2.0 14.0) | 6.0 (2.0 15.0) | 5.0 (2.0 14.0) | 5.0 (2.0 14.0) | 5.0 (2.0 14.0) | 6.0 (2.0 13.0) | 5.0 (2.0 13.0) | 5.0 (2.0 13.0) | 0.612 |
|  |  |  |  |  |  |  |  |  |  |  |  |  |
